# Enrichment of Variations in KIR3DL1/S1 and KIR2DL2/L3 among H1N1/09 ICU Patients: An Exploratory Study

**DOI:** 10.1371/journal.pone.0029200

**Published:** 2011-12-28

**Authors:** David La, Chris Czarnecki, Hani El-Gabalawy, Anand Kumar, Adrienne F. A. Meyers, Nathalie Bastien, J. Neil Simonsen, Francis A. Plummer, Ma Luo

**Affiliations:** 1 National Microbiology Laboratory, Public Health Agency of Canada, Winnipeg, Manitoba, Canada; 2 Department of Medical Microbiology, University of Manitoba, Winnipeg, Manitoba, Canada; 3 Department of Internal Medicine, University of Manitoba, Winnipeg, Manitoba, Canada; 4 Section of Critical Care Medicine, Health Sciences Centre and St. Boniface Hospital, University of Manitoba, Winnipeg, Canada; Karolinska Institutet, Sweden

## Abstract

**Background:**

Infection by the pandemic influenza A (H1N1/09) virus resulted in significant pathology among specific ethnic groups worldwide. Natural Killer (NK) cells are important in early innate immune responses to viral infections. Activation of NK cells, in part, depend on killer-cell immunoglobulin-like receptors (KIR) and HLA class I ligand interactions. To study factors involved in NK cell dysfunction in overactive immune responses to H1N1 infection, KIR3DL1/S1 and KIR2DL2/L3 allotypes and cognate HLA ligands of H1N1/09 intensive-care unit (ICU) patients were determined.

**Methodology and Findings:**

KIR3DL1/S1, KIR2DL2/L3, and HLA -B and -C of 51 H1N1/09 ICU patients and 105 H1N1-negative subjects (St. Theresa Point, Manitoba) were characterized. We detected an increase of 3DL1 ligand-negative pairs (3DL1/S1^+^ Bw6^+^ Bw4^−^), and a lack of 2DL1 HLA-C2 ligands, among ICU patients. They were also significantly enriched for 2DL2/L3 ligand-positive pairs (*P*<0.001, *Pc*<0.001; Odds Ratio:6.3158, CI95%:2.481–16.078). Relative to St. Theresa aboriginals (STh) and Venezuelan Amerindians (VA), allotypes enriched among aboriginal ICU patients (Ab) were: 2DL3 (Ab>VA, *P* = 0.024, *Pc* = 0.047; Odds Ratio:2.563, CI95%:1.109–5.923), 3DL1*00101 (Ab>VA, *P*<0.001, *Pc*<0.001), 3DL1*01502 (Ab>STh, *P* = 0.034, *Pc* = 0.268), and 3DL1*029 (Ab>STh, *P*  = 0.039, *Pc* = 0.301). Aboriginal patients ligand-positive for 3DL1/S1 and 2DL1 had the lowest probabilities of death (R_d_) (R_d_ = 28%), compared to patients that were 3DL1/S1 ligand-negative (R_d_ = 52%) or carried 3DL1*029 (R_d_ = 52%). Relative to Caucasoids (CA), two allotypes were enriched among non-aboriginal ICU patients (NAb): 3DL1*00401 (NAb>CA, *P*<0.001, *Pc*<0.001) and 3DL1*01502 (CA<NAb, *P* = 0.012, *Pc* = 0.156). Non-aboriginal patients with ligands for all three KIRs (3DL1/S1, 2DL2/L3, and 2DL1) had the lowest probabilities of death (R_d_ = 36%), compared to subjects with 3DL1*01502 (R_d_ = 48%) and/or 3DL1*00401 (R_d_ = 58%).

**Conclusions:**

Specific KIR3DL1/S1 allotypes, 3DL1/S1 and 2DL1 ligand-negative pairs, and 2DL2/L3 ligand-positive pairs were enriched among ICU patients. This suggests a possible association with NK cell dysfunction in patients with overactive immune responses to H1N1/09, leading to severe disease.

## Introduction

The recent pandemic influenza A (H1N1/09) virus, relative to seasonal influenza, was observed to have higher transmissibility, albeit with lower mortality rates [1], [2]. Although there were more than 622,482 reported cases worldwide as of November 2009 [3], [4], overactive immune responses to H1N1/09 infections leading to significant pathology were reported to be highly prevalent among youths and young adults. Recently, the basis for this observation was suggested to be a lack of antigenic site recognition for the hemagglutinin structure among those age groups [5], [6]. Nonetheless, those of specific ethnic backgrounds were also observed to be disproportionately affected, namely aboriginal and minority groups [7]–[11]. Although the factors behind the disproportionate impact of H1N1/09 remain to be fully elucidated, there is some evidence to suggest a role for natural killer (NK) cells in the control of influenza viral loads.

The importance of NK cells was suggested in a recent study, where NK cell frequencies were found to be significantly reduced in patients with severe responses to H1N1/09 infections, relative to mild cases and healthy controls [12]. On the other hand, CD8^+^ effector T cells, regardless of patient disease status, were detected at normal levels. A study of NK cells in infected mice also showed that reduced NK cell activity in the earlier stages led to significantly increased viral growth [13].

NK cells function as the primary innate immune response against viruses and tumours and are capable of inducing stimulatory and regulatory effects on the adaptive immune response [14]–[16]. Their functions are determined by a broad array of activating and inhibitory receptors [17]. Killer-cell immunoglobulin-like receptors (KIR) are a large family of receptors comprised mainly of those with inhibitory capacities that recognize HLA Class I ligands [18], [19], along with a few variants that possess activating functions. In the context of all known KIR variants in humans, the KIR3DL1/S1 subset is widespread and encode for both an inhibitory (3DL1) and activating (3DS1) receptor [19]. KIR3DL1 receptors recognize class I HLA-B proteins that carry a Bw4 motif [20], while HLA-B Bw4-80I proteins are the putative ligands of KIR3DS1 [21]. Analogous inhibitory KIR subsets, such as KIR2DL1 and KIR2DL2/L3, recognize HLA-C2 and HLA-C1 ligands, respectively [22], [23]. Given the independent diversities of KIR and HLA, there likely exist combinations which can variably influence the efficacy of NK cell responses in the control of viral infections due to the presence of certain NK cell receptor/ligand pairs [24]–[26].

To date, there have been no published reports pertaining to KIR receptors and their ligands, in relation to NK cell dysfunction, in severe cases of H1N1/09 infections. Identification of potential associations of specific KIR allotypes and their ligands with severe responses to H1N1/09 infections may assist in the predetermination of populations most at risk via information on frequencies of molecular immune response allotypes. In this study, we compared H1N1/09 ICU patients with H1N1-negative subjects from St. Theresa Point and similar world populations. We investigated KIR3DL1/KIR3DS1 and KIR2DL2/KIR2DL3 in this study, as each pair share the same locus, encode for both inhibitory and/or activating receptors, and their HLA ligands are highly polymorphic [27].

## Results

### KIR3DL1/S1 allotype and KIR2DL2/L3 frequencies of H1N1/09 ICU patients

The KIR3DL1/S1 and KIR2DL2/L3 frequencies of ICU patients are summarized in Table 1. In total, fourteen 3DL1 and two 3DS1 allotypes were detected. 3DS1*01301 was the most prevalent (26.5%), followed by 3DL1*00101(15.7%), 3DL1*01502(15.7%), 3DL1*00401 (9.8%), and 3DL1*00501(8.8%). Three allotypes, 3DS1*01301, 3DL1*00101, and *01502, were found to account for 70% of allotypes among ICU patients of aboriginal descent (Ab). Greater diversity was found among patients of non-aboriginal descent (NAb), as five allotypes accounted for a combined frequency of 77%: 3DS1*01301, 3DL1*00401, *00101, *01502, *002.

**Table 1:** KIR3DL1/S1 allele frequencies of ICU patients and St. Theresa aboriginals.

	ICU Patients, C								
	All (2n = 102)		Ab (2n = 40)		NAb (2n = 62)		STh (2n = 210)		*P* (*Pc*)
KIR Allele	2n	AF	2n	AF	2n	AF	2n	AF	*Ab vs STh*
3DL1*00101	16	15.7%	8	20.0%	8	12.9%	46	21.9%	0.486
3DL1*002	7	6.9%	0	0.0%	7	11.3%	1	0.5%	1.000
3DL1*00401	10	9.8%	0	0.0%	10	16.1%	0	0.0%	-
3DL1*00402	2	2.0%	0	0.0%	2	3.3%	0	0.0%	-
3DL1*00501	9	8.8%	3	7.5%	6	9.7%	38	18.1%	0.108
3DL1*007	4	3.9%	1	2.5%	3	4.8%	0	0.0%	0.163
3DL1*008	1	1.0%	0	0.0%	1	1.6%	0	0.0%	-
3DL1*009	1	1.0%	0	0.0%	1	1.6%	0	0.0%	-
3DL1*01501	0	0.0%	0	0.0%	0	0.0%	0	0.0%	-
3DL1*01502	16	15.7%	8	20.0%	8	12.9%	16	7.6%	**0.034 (0.268)** 1
3DL1*019	0	0.0%	0	0.0%	0	0.0%	0	0.0%	-
3DL1*020	1	1.0%	0	0.0%	1	1.6%	0	0.0%	-
3DL1*029	4	3.9%	4	10.0%	0	0.0%	5	2.4%	**0.039 (0.301)** 2
3DL1*051	1	1.0%	1	2.5%	0	0.0%	1	0.5%	0.300
3DS1*010	3	2.9%	3	7.5%	0	0.0%	5	2.4%	0.119
3DS1*01301	27	26.5%	12	30.0%	15	24.2%	98	46.7%	0.055
3DL1	72	70.6%	25	62.5%	47	75.8%	107	51.0%	0.227
3DS1	30	29.4%	15	37.5%	15	24.2%	103	49.0%	
2DL2	22	21.6%	7	17.5%	15	24.2%	31	14.8%	0.635
2DL3	80	78.4%	33	82.5%	47	75.8%	179	85.2%	

AF, allele frequency; Ab, Aboriginal; NAb, Non-aboriginal.

STh, St. Theresa aboriginals; C, H1N1 confirmed cases.

1 Odds Ratio:3.031(CI95%:1.199–7.663);

2 Odds Ratio:4.556(CI95%:1.167–17.779).

KIR2DL2/L3 were detected in all patients, with a high number of patients carrying only 2DL3 allotypes (Ab, 70%; NAb, 54.8%). Patients with only 2DL2 were rare among both ICU subgroups (Ab, 5%; NAb, 3.2%). These results were consistent with another study on First Nations KIR gene profiles [28]. Differences in 2DL2/L3 distributions between Ab and St. Theresa Point (STh) subjects were not statistically significant.

### Enrichment of KIR3DL1/S1 allotypes in H1N1/09 ICU patients of aboriginal descent

In order to further characterize the diversity and distribution of allotypes among Ab patients, we compared those with high frequencies to St. Theresa Point subjects. This was a viable group for comparison, as STh subjects were highly susceptible to severe responses during the first wave of the pandemic [29], [30]. Because aboriginal ICU patient samples were derived from hospitals across the country, they were assumed to be more ethnically diverse than St. Theresa aboriginals. This was confirmed when their distributions of HLA-B and C were compared.

Nine different allotypes were detected between Ab and STh (Table 1). 3DS1*01301 and 3DL1*00101 were especially widespread among both groups. Similar to Ab patients, a lack of diversity was detected among the STh subjects, as three allotypes (3DS1*01301, 3DL1*00101, and *00501) accounted for a combined frequency of 86.7%.

Prior to correction for multiple comparisons, two allotypes were found to be significantly enriched among Ab patients: 3DL1*01502 (Ab>STh, *P* = 0.034, *Pc* = 0.268; Odds Ratio:3.031, CI95%:1.199–7.663) and 3DL1*029 (Ab>STh, *P* = 0.039, *Pc* = 0.301; Odds Ratio:4.556, CI95%:1.167–17.779). 3DS1*010 was detected at a higher frequency in Ab patients, but the difference was not statistically significant (Ab>STh, *P* = 0.119).

### Lack of KIR3DL1/S1 and HLA-B Bw4 ligand pairs in H1N1/09 ICU patients

In the absence of cognate ligands, the presence and function of KIR3DL1/S1 receptors are deemed negligible, due to their inability to elicit a relevant response within NK cells [18], [20], [21]. Subjects detected as KIR3DL1/S1^+^ HLA-B Bw6^+^ Bw4^−^ were considered to be ligand-negative. To determine the relative degree of ligand-negative pairs present among ICU patients, we determined the prevalence of 3DL1/S1 and their cognate ligands (Bw4^+^), among the ICU patients and St. Theresa subjects. Overall, compared to STh subjects, 3DL1 ligand-negative pairs were found to be proportionally higher in H1N1/09 ICU patients (ICU>STh, *P* = 0.093) (Table 2), albeit not statistically different due to the small sample size. In contrast, 3DL1/S1-Bw4^+^ pairs were common among the healthy STh subjects (81%). Of the highly prevalent 3DL1/S1 allotypes that were common among both groups, the frequencies of several ligand-negative pairs (3DL1*00101, 3DL1*01502, 3DS1*010, and 3DS1*01301) were much higher among the H1N1/09 patient groups.

**Table 2:** Comparison of KIR3DL1/S1 allotypes in the presence or absence of their ligands within H1N1- confirmed ICU patients and St. Theresa aboriginals.

	ICU patients, Confirmed		
KIR Allele	Ab	Nab	STh
3DL1*00101			
*Bw4^+^*	5 (71.4%)	5 (62.5%)	37 (86.0%)
*Bw6^+^, Bw4^−^*	2 (28.6%)	3 (37.5%)	6 (14.0%)
3DL1*002			
*Bw4^+^*	0 (0%)	4 (66.7%)	1 (100%)
*Bw6^+^, Bw4^−^*	0 (0%)	2 (33.3%)	0 (0%)
3DL1*00501			
*Bw4^+^*	2 (66.7%)	4 (66.7%)	25 (69.4%)
*Bw6^+^, Bw4^−^*	1 (33.3%)	2 (33.3%)	11 (30.6%)
3DL1*01502			
*Bw4^+^*	4 (57.1%)	5 (71.4%)	15 (93.7%)
*Bw6^+^, Bw4^−^*	3 (42.9%)	2 (28.6%)	1 (6.3%)
3DS1*10			
*Bw4^+^*	1 (33.3%)	0 (0%)	5 (100%)
*Bw6^+^, Bw4^−^*	2 (66.7%)	0 (0%)	0 (0%)
3DS1*01301			
*Bw4^+^*	9 (90.0%)	9 (75.0%)	56 (77.8%)
*Bw6^+^, Bw4^−^*	1 (10.0%)	3 (25.0%)	16 (22.2%)
3DL1			
*Bw4^+^*	13 (72.2%)	18 (64.3%)	63 (79.8%)
*Bw6^+^, Bw4^−^*	5 (27.8%)	10 (35.7%)	16 (20.3%)
3DS1			
*Bw4^+^*	10 (76.9%)	9 (75.0%)	61 (79.2%)
*Bw6^+^, Bw4^−^*	3 (23.1%)	3 (25.0%)	16 (20.8%)
3DL1*			
*Bw4^+^*	31 (67.4%)		85 (81.0%)
*Bw6^+^, Bw4^−^*	15 (32.6%)		20 (19.0%)
2DL2			
*C1^+^*	4 (66.7%)	14 (100%)	14 (45.2%)
*C1^−^, C2^+^*	2 (33.3%)	0 (0%)	17 (54.8%)
2DL3			
*C1^+^*	13 (68.4%)	30(100%)	57 (54.3%)
*C1^−^, C2^+^*	6 (31.6%)	0 (0%)	48 (45.7%)
2DL2/L3**			
*C1^+^*	45 (88.2%)		57 (54.3%)
*C1^−^, C2^+^*	6 (11.8%)		48 (45.7%)

Ab, Aboriginal; NAb, Non-aboriginal; STh, St. Theresa aboriginals.

**P<0.001, *Pc*<0.001.

*P<0.10.

### Enrichment of KIR2DL2/L3 and HLA-C C1 ligand pairs among H1N1/09 ICU patients

In order to further determine the frequency of other functional KIR allotype-ligand relationships, the distribution of KIR2DL2/L3 and HLA-C1/C2 were determined. Subjects with 2DL2/L3^+^ C1^+^ C2^+/−^ were considered to be ligand-positive (Table 2). All NAb patients were ligand-positive. Compared to STh subjects, ligand-positive pairs were significantly higher in H1N1/09 ICU patients overall (*P*<0.001, *Pc*<0.001; Odds Ratio:6.3158, CI95%:2.481–16.078). Conversely, there was a significant lack of C2 allotypes (KIR2DL1 ligands) among NAb patients, as compared to both Ab and STh subjects (NAb (12.9%)<Ab (50.0%)<STh (68.1%), *P*_NAbvsAb_<0.001, *Pc*<0.001; *P*_NAbvsSTh_<0.001, *Pc*<0.001).

### Comparison of KIR3DL1/S1 and KIR2DL2/L3 frequencies between H1N1/09 ICU subgroups and world populations

During the initial waves of the H1N1/09 pandemic, several populations were disproportionately affected by overactive immune responses to infections. We compared the frequencies of KIR3DL1/S1 allotypes enriched among the ICU subgroups (Ab and NAb) to that of analogous populations in the world; the Venezuelan Amerindians (VA) and Caucasoids (CA) of various countries [31]. The VA group consisted of three Amerindian tribes: the Yucpa, Bari, and Warao [32]. The CA group consisted mainly of six different populations: England, Georgia, Spain, Turkey, USA California, and USA European [33], [34].

The Amerindian tribes were similarly found to lack diversity in 3DL1/S1 and their most prevalent allotypes were 3DS1*01301, 3DL1*005, and *01502 (Table 1, 3). 3DL1*00101, common to both Ab and STh subjects, was absent in VA subjects (VA<Ab, *P*<0.001, *Pc*<0.001; Odds Ratio:infinite). In Ab patients, KIR2DL2 and KIR2DL3 allotypes were found to be significantly reduced and enriched, respectively, in comparison to VA subjects (Ab>VA, *P* = 0.024, *Pc* = 0.047; Odds Ratio:2.563, CI95%:1.109–5.923).

**Table 3:** Comparison of KIR3DL1/S1 allele frequencies between ICU patients and analogous world populations.

	ICU Patients, C		Ethnic Groups		*P* (*Pc*)	
KIR Allele	Ab	NAb	Va^a^^,^ b	Ca^a^^,^ c	*Ab vs VA*	*NAb vs CA*
3DL1*001	0.200	0.129	0.000±	0.162±	**<0.001 (<0.001)** 1	0.603
3DL1*002	0.000	0.113	0.000	0.120	-	1.000
3DL1*00401	0.000	0.161	0.002	0.034	1.000	**<0.001 (<0.001)** 2
3DL1*00402	0.000	0.033	0.000	0.119	-	0.029
3DL1*005	0.075	0.097	0.128±	0.145±	0.455	0.841
3DL1*007	0.025	0.048	0.002	0.033	0.154	0.167
3DL1*008	0.000	0.016	0.005	0.051	0.222	1.000
3DL1*009	0.000	0.016	0.000	0.020	-	0.562
3DL1*01501	0.000	0.000	0.000	0.004	-	1.000
3DL1*01502	0.200	0.129	0.373	0.064	0.038	**0.012 (0.156)** 3
3DL1*019	0.000	0.000	0.000	0.001	-	1.000
3DL1*020	0.000	0.016	0.000	0.008	-	0.386
3DL1*029	0.100	0.000	0.065	0.001	0.336	1.000
3DS1*01301	0.300	0.242	0.407	0.233	0.239	0.878
3DL1	0.625	0.758	0.578	0.802^d^	0.619	0.424
3DS1	0.375	0.242	0.422	0.198^d^		
2DL2	0.175	0.242	0.352	0.320^d^	**0.024 (0.047)** 4	0.220
2DL3	0.825	0.758	0.648	0.680^d^		

Ab, Aboriginal; NAb, Non-aboriginal; VA, Venezuelan Amerindian; CA, Caucasoid.

3DL1*051, 3DS1*010 comparisons not available.

a Allele frequencies derived from Allele frequency net (28).

b 2n = 460;

c 2n = 4056;

d 2n = 17,680.

± Frequencies derived from low-resolution data.

1 Odds Ratio:infinite;

2 Odds Ratio:5.460(CI95%:2.717–10.970);

3 Odds Ratio:2.163(CI95%:1.019–4.593);

4 For 2DL2, Odds Ratio:0.390(CI95%:0.169–0.902); For 2DL3, Odds Ratio:2.563(CI95%:1.109–5.923).

World Caucasoids were highly diversified for 3DL1/S1 (Table 3). Eighty-seven percent of NAb ICU patients were Caucasoids and when compared to the CA group, 3DL1*00401 was significantly found to be enriched (CA<NAb, *P*<0.001, *Pc*<0.001; Odds Ratio:5.460, CI95%:2.717–10.970). Prior to correction for multiple comparisons, 3DL1*01502 was also markedly enriched in NAb (CA<NAb, *P* = 0.012, *Pc* = 0.156; Odds Ratio:2.163, CI95%:1.019–4.593). Differences in 2DL2/L3 between NAb patients and the CA group were not statistically significant.

### APACHE II score comparison between H1N1/09 ICU subgroups and allotype/ligand pairs

APACHE II is a severity of disease classification system, commonly used for ICU patients, which calculates risk of hospital death on the basis of twelve routine physiologic measurements and their degree of divergence from typical thresholds [35]. Total APACHE II scores were calculated for all H1N1/09 ICU patients and probabilities of death (R_d_) were determined (Table 4). Because KIR3DS1 has only been shown to bind to Bw4-80I ligands [26], 3DS1^+^/3DL1^−^ Bw4-80T^+^ 80I^−^ pairs were considered to be ligand-negative, when correlating allotype-ligand pairs with APACHE II scores.

**Table 4:** Comparison of average probabilities of death among H1N1 ICU patients based on APACHE II severity of disease classification system.

	Aboriginal		Non-Aboriginal	
Allele/Ligand	n	Death Rate (%)	n	Death Rate (%)
2DL2/L3 C1^het^	8 (40%)	36	9 (29%)	40
2DL2/L3 C1^hom^	6 (30%)	43	22 (71%)	40
2DL2/L3 C2^hom^	6 (30%)	38	-	-
2DL2/L3 C1^+^	14 (70%)	39	9 (29%)	40
2DL2^+^ C1^+^	3 (15%)	21	12 (39%)	38
2DL2^+^ 2DL3^−^ C1^+^	10 (50%)	46	19 (61%)	42
3DL1/S1 Bw4^het^	10 (50%)	29	15 (48%)	41
3DL1/S1 Bw4^hom^	4 (20%)	41	2 (7%)	48
3DL1/S1 Bw6^hom^	6 (30%)	52	11 (36%)	39
3DL1/S1 Bw4^+^	14 (70%)	34	17 (55%)	43
Bw4^+^ and C1^−^	5 (25%)	35	-	-
Bw4^−^ and C1^+^	5 (25%)	52	10 (32%)	38
Bw4^+^ and C2^+^ C1^+/−^	9 (45%)	28	6 (19%)	36
Bw4^−^ and C2^+^ C1^+/−^	4 (20%)	51	1 (3%)	55
Bw4^+^ and C1^+^ C2^+^	5 (25%)	27	6 (19%)	36
3DL1*00101 Bw4^+^	5 (25%)	40	5 (16%)	37
3DL1*01502 Bw4^+^	4 (20%)	33	5 (16%)	48
3DL1*029 Bw4^+^	3 (15%)	52	-	-
3DL1*00401 Bw4^+^	-	-	5 (16%)	58
3DS1*01301 Bw4-80I^+^	9 (45%)	32	8 (26%)	48

het, heterozygous; hom, homozygous.

Among Ab patients, the mean probability of death was 38% (Range: 8%–75%). Low death rates were consistently observed in subjects that were 3DL1/S1 Bw4^+^ ligand-positive and in combination with HLA-C2 (R_d_ = 28%). No notable differences were found based solely on the presence of 2DL2/L3 C1^+/−^ pairs. Of the 3DL1 allotypes enriched in aboriginal patients, only 3DL1*029, which was rare in most ethnic groups with exception to the Yucpa Amerindians from Venezuela [31], was associated to a higher probability of death (R_d_ = 52%). Because of the variations in binding affinity of 2DL2 and 2DL3 to HLA-C1 [36], the disease severities between patients with these allotypes were compared. Differences in death rates were found between 2DL2^+^ (R_d_ = 21%) and 2DL3^+^ 2DL2^−^ patients (R_d_ = 46%).

Non-aboriginal ICU patients had slightly higher probabilities of death with a mean of 40% (Range: 8%–80%). All NAb patients were 2DL2/L3 C1^+^ ligand-positive. Contrary to Ab patients, low probabilities of death (R_d_ = 39%) were found in 3DL1/S1 Bw6^+^ ligand-negative pairs. However, a comparable death rate (R_d_ = 36%) was also observed in 3DL1/S1 Bw4^+^ HLA-C2^+^ subjects, which may indicate either a lack of effect by 3DL1/S1 on disease severity in non-aboriginals, or confounding due to an absence of 2DL2/L3 C1^+^ ligand-negative patients. Patients with ligands for all three KIRs (2DL1, 2DL2/L3, and 3DL1/S1) had the lowest probability of death (R_d_ = 36%). 3DL1*01502 and 3DL1*00401, allotypes enriched in non-aboriginal patients, were correlated with high disease severity (R_d_ = 48%; and R_d_ = 58%, respectively). There were no significant differences in disease severity between 2DL2^+^ (R_d_ = 38%) and 2DL3^+^ 2DL2^−^ (R_d_ = 42%) patients.

## Discussion

The objective of this study was to identify KIR3DL1/S1 and KIR2DL2/L3 allotypes, and corresponding ligands, enriched in aboriginal and non-aboriginal H1N1/09 ICU patients from the initial 2009 H1N1 pandemic waves. The premise was that specific allotypes and/or ligand combinations could predispose patients towards severe responses to H1N1/09 infections. In a general population, only a small proportion of individuals will develop such overactive immune responses [37]–[39]. The enrichment of specific KIR allotypes was evident when viewed in comparison to an H1N1-negative control population, St. Theresa Point; a First Nations community that was severely impacted during the first and second wave of the pandemic [29], [30]. Specific KIR allotype enrichment was also present, relative to Amerindians and world Caucasoids. Despite the small sample size of the ICU patients, several significant differences between groups were observed. As this is an exploratory study, further confirmation of the findings in future studies will be necessary.

In general, patients and populations of aboriginal descent were all found to lack 3DL1/S1 diversity, relative to world Caucasoids and ICU non-aboriginal patients. Although nine allotypes were detected, four allotypes accounted for the majority among all aboriginal subjects. This relatively low degree of diversity may nonetheless confer a greater susceptibility to infectious diseases, in general [11]. Additionally, 2DL3 allotypes were prevalent among ICU and St. Theresa subjects, in addition to a lack of 2DL2/2DL2 (homozygous) allotype carriers, especially among St. Theresa subjects. Enrichment of 2DL3 was also detected in aboriginal patients, in comparison to Venezuelan Amerindians. However, the 2DL2/L3 distributions between all other groups were similar and were consistent with the report from a study on KIR profiles in First Nations and Caucasoids of European Descent [28].

Highly expressed allotypes of KIR3DL1 (3DL1*001, *002, 01502), defined by their expression levels and/or inhibitory capacities [40]–[42], were common to both healthy St. Theresa and H1N1/09 ICU subjects. Yet among the ICU patients, these allotypes were frequently found ligand-negative. Overall, there was a larger proportion of 3DL1 ligand-negative ICU patients, relative to St. Theresa subjects. Although presumptive, it is highly possible then that, in conjunction with their cognate ligands, KIR3DL1 may play a partial role in preventing infection or inhibiting the development of severe responses.

Conversely, the significant enrichment of 2DL2/L3 ligand-positive pairs in ICU patients, in comparison to St. Theresa subjects, may imply that 2DL2/L3-ligand interactions contribute to greater disease severity. APACHE II scores (probability of hospital death) and available KIR/ligand data further suggested this possibility. Interestingly, correlations between disease severity and absence of 3DL1/S1 ligands and 2DL2 allotypes, were evident in aboriginal ICU patients only. The lack of clarity among non-aboriginal patients may be due to a lack of C2 ligands that was specifically observed in non-aboriginal ICU patients, and the expected high prevalence of their corresponding 2DL1 allotypes in both First Nations and Caucasoids of European descent [28], [31]. Nonetheless, the small proportion of non-aboriginal patients with ligands for all three KIR subtypes had the least severe responses.

Taken together, severe responses to H1N1/09, among other factors, may be dependent on 3DL1/S1, 2DL1, and 2DL2 ligand interactions, at least in the case of aboriginal patients. On the other hand, non-aboriginal patients may be affected by specific 3DL1 allotypes and a lack of 2DL1 allotype-ligands interactions. In future studies, it would be interesting to determine the distribution of specific 2DL1 and 2DL2/L3 allotypes, in conjunction with their ligands, for a larger sample of H1N1/09 ICU patients. This would allow for further clarification and characterization of the function, or lack thereof, of NK cells in patients with severe responses.

Lastly, marked differences were found in the frequencies of two synonymous allotypes, 3DL1*00401 and 3DL1*00402, between non-aboriginal ICU patients and Caucasoids. Although highly speculative, the mutations could produce two different proteins, possibly via changes in mRNA stability or alternative splice sites [43], [44]. Their phenotypic effects, if any, will need to be further investigated.

In summary, among ICU patients with severe responses to H1N1/09, 3DL1*00101, 3DL1*01502, and 3DL1*029, were enriched in aboriginal ICU patients, while 3DL1*00401 and 3DL1*01502 were enriched in non-aboriginals ICU patients. Likewise, the ligand-negative pairs KIR3DL1/S1^+^ Bw6^+^ Bw4^−^ and KIR2DL1 C2^−^ C1^+^, and ligand-positive pair KIR2DL3 C1^+^, were also observed to be proportionally higher in ICU patients, relative to healthy St. Theresa controls. As such, the study shows that the enrichment of specific allotypes and a disproportional distribution of cognate HLA class I ligands are likely factors that mediated NK cell dysfunction and lead to the development of severe responses to H1N1/09 in ICU patients.

## Materials and Methods

### Study Population

The study used data from two separate cohorts with a total of 156 subjects (125 Aboriginal, 27 Caucasoid, 2 South Asian, and 2 unknown/mix ethnics). The first cohort consisted of 51 H1N1/09 intensive-care unit (ICU) patients with severe cases of infection. The second included 105 aboriginal H1N1-negative subjects from St. Theresa Point. Screening of the St. Theresa Point population was undertaken by analyzing DNA samples obtained in 2007–09, in the context of a study evaluating the prevalence of rheumatoid arthritis (RA) risk factors in the community. Individuals aged 18–55 were randomly selected from the community at large based on their willingness to participate in the RA study. All ICU patients were H1N1-confirmed by RT-PCR testing.

#### Ethics Statement

Permission to use the St. Theresa Point samples for the H1N1/09 study was specifically obtained from the Research Ethics Board of the University of Manitoba and from the leadership of the community through the Band Council. The study of ICU patients was approved using a full consent process through the Research Ethics Board of the University of Manitoba. The data were analyzed anonymously.

### KIR3DL1/S1 and HLA Genotyping

Genomic DNA was isolated from whole blood samples that were stored in PAXgene Blood RNA tubes (PreAnalytix) using a QIAmp DNA Mini Kit and the EZ1 BioRobot (QIAgen Inc, Mississauga, Ontario, Canada).

#### PCR amplification

KIR3DL1/S1 exons 1–5, 7, and 9, KIR2DL2/3 exon 4 [45], and HLA-B exons 2 and 3 [46] were amplified using genomic DNA with gene-specific primers (Table 5). PCR amplifications were confirmed by 1% agarose gel electrophoresis with ethidium bromide. PCR products were purified using Agencourt AMPure XP Kits (Beckman Coulter) and resuspended in Tris-EDTA buffer (pH 8.0).

**Table 5:** Amplification and sequencing primers for KIR3DL1/S1 typing.

Name	Specificity	Primer Sequence (5′ 3′)	Location	Annealing Temperature
L1S1-1FPCR	5′ Exon 1	CGAGGTGTCAATTCTAGTGAGAG	Intron 1	61 to 51°C^c^
L1S1-1RPCR	3′ Exon 1	CACTTCAGGCCCATAACTCCAC	Intron 2	61 to 51°C^c^
L1S1-1FSEQ	Exon 1	CGAGGTGTCAATTCTAGTGAGAG	Intron 1	53.0°C
L1S1-1RSEQ	Exon 1	CTAGGCCCATATCTTTACCTCC	Intron 2	55.0°C
L1S1-2-3FPCR	5′ Exon 2	GAGATCCTTGTTCCTGGGG	Intron 2	56.2°C
L1S1-2-3RPCR	3′ Exon 3	CGTCTCCCTCCCACTACAC	Intron 3	56.2°C
L1S1-2FSEQ	Exon 2	CAGCGAGGGTGAGTTTAC	Intron 2	56.9°C
L1S1-2RSEQ	Exon 2	GAGGGTCCCCTCTTCCTAGTG	Intron 3	55.0°C
L1S1-3FSEQ	Exon 3	GTGGAAATGGGGAGAATCTTCTGG	Intron 3	55.0°C
L1S1-3RSEQ	Exon 3	CAGAAGCTCTGGGATTCAG	Intron 4	58.0°C
L1S1-4FPCR	5′ Exon 4	CATGCAGCCTGTCCTCTTC	Intron 4	57.6°C
L1S1-4RPCR	3′ Exon 4	ACACGGCATCTGTAGGTGG	Intron 5	57.6°C
L1S1-4FSEQ	Exon 4	GGGAGGAGAGAGACAGACACG	Intron 4	53.0°C
L1S1-4RSEQ	Exon 4	CAGACCTCACCAAGTCAC	Intron 5	52.0°C
L1S1-5FPCR	5′ Exon 5	GACAGAGAGGCAGACAGAGAGG	Intron 5	64 to 54°C^c^
L1S1-5RPCR	3′ Exon 5	CTGACTCCGCCCTCACACCTG	Intron 6	64 to 54°C^c^
L1S1-5FSEQ	Exon 5	GAGAGAGAGAGAGAGAGCATTAG	Intron 5	55.0°C
L1S1-5RSEQ	Exon 5	CTCTGCATCTGTCCATGCTTTTC	Intron 6	55.0°C
L1S1-7-9FPCR^a^	5′ Exon 7	GCTATAACTGAGAAAGCAGGAGG	Intron 7	64 to 54°C^c^
L1S1-7-9RPCR^a^	3′ Exon 9	CATTTGTAAGCAAGWGAGAGGCAC	Intron 9	64 to 54°C^c^
L1S1-7FSEQ^a^	Exon 7	GGGTGCTTGTCCKAAAGAGAYGC	Intron 7	58.9°C
L1-9FSEQ^a^^,^ b	Exon 9	CACTCAGCATTTCCCTCCCTCAC	Intron 9	54.4°C
L1-9RSEQ^a^^,^ b	Exon 9	GGCTGTTGTCTCCCTAGAAGACG	Intron 10	58.0°C

a Does not amplify 3DL1*059, 3DL1*060, 3DL1*061.

b 3DL1 only.

c Touchdown PCR temperature range.

#### Sequencing

PCR products were sequenced using BigDye™ Terminator Cycle Sequencing Kits (Applied Biosystems) V1.1. Purified PCR products were analyzed using an ABI PRISM 3130×l Genetic Analyzer (Applied Biosystems) and genotyped using Codon Express™, a taxonomy-based sequencing analysis software, with the KIR and HLA databases from IMGT/HLA and IPD/KIR, respectively [47]–[49].

### Statistical Analysis

Frequency analysis was performed using SPSS 13.0 for Windows. The statistical significance of difference between allele frequencies were calculated using Fisher's Exact Test for small numbers via Microsoft Research and VassarStats [50], [51]. Correct *P* values (*Pc*) were obtained using the Sidak method by calculating 1-(1- *P*)^n^, where n is the number of alleles analyzed in each group. A *P* value of less than 0.05 was considered significant.
